# Noise Response Data Reveal Novel Controllability Gramian for Nonlinear Network Dynamics

**DOI:** 10.1038/srep27300

**Published:** 2016-06-06

**Authors:** Kenji Kashima

**Affiliations:** 1 Graduate School of Informatics, Kyoto University, Kyoto 606-8501, Japan

## Abstract

Control of nonlinear large-scale dynamical networks, e.g., collective behavior of agents interacting via a scale-free connection topology, is a central problem in many scientific and engineering fields. For the linear version of this problem, the so-called controllability Gramian has played an important role to quantify how effectively the dynamical states are reachable by a suitable driving input. In this paper, we first extend the notion of the controllability Gramian to nonlinear dynamics in terms of the Gibbs distribution. Next, we show that, when the networks are open to environmental noise, the newly defined Gramian is equal to the covariance matrix associated with randomly excited, but uncontrolled, dynamical state trajectories. This fact theoretically justifies a simple Monte Carlo simulation that can extract effectively controllable subdynamics in nonlinear complex networks. In addition, the result provides a novel insight into the relationship between controllability and statistical mechanics.

Control, i.e., external forcing aimed at achieving desirable dynamical trajectories, of nonlinear large-scale dynamical networks is of major interest in many research fields such as gene regulatory networks, infection spreads, human brains, financial markets, smart grids, to list just a few^1^,^2^,^3^,^4^. To investigate how difficult such networks are to control, controllability, originally defined in control theory^5^,^6^, has attracted much attention, mainly in physics research^1^,^2^,^7^,^8^,^9^,^10^,^11^,^12^,^13^,^14^,^15^. Among them, Kalman’s controllabilty matrix has played an important role to determine whether every dynamical state of a linear system is reachable^1^. Beyond this controllability *determination*, the so-called controllability Gramian, that is only defined for linear systems, provides much of the *quantitative* information concerning this problem. For example, every dynamical state is reachable if and only if the Gramian is nonsingular. Moreover, the minimum control energy required to drive the current state to a target one is represented as a quadratic form associated to the inverse of the Gramian, which is utilized to analyze the effect of connection topology^7^. In this context, the condition number of the Gramian is a meaningful index to characterize the nonlocality of linear complex networks^8^.

The controllability of complex networks with *nonlinear* dynamics is also being actively investigated^16^,^17^. The Lie bracket gives a natural extension of Kalman’s controllability matrix rank condition for the controllability determination^16^. However, an analogous controllability Gramian for nonlinear dynamics has not yet been developed, even in control theory^18^,^19^,^20^,^21^,^22^,^23^, although the controllability Gramian of a linearized system is useful in some applications. One of only a few existing approaches is the empirical Gramian^24^ that appears in simulation-based model reduction methods^25^,^26^ mainly developed in computational physics and numerical analysis. The empirical Gramian is constructed using simulation data, which is in stark contrast to the controllability Gramian. Furthermore, it has been widely applied to *nonlinear* large-scale systems^24^,^27^. However, although this is equal to the controllability Gramian when the dynamics are linear, there are no theoretical underpinnings for such an application to nonlinear cases.

The goal of this paper is to introduce a novel matrix measure for the controllability quantification of nonlinear network dynamics, to reveal its specific feature under stochastic noise, and to provide a simulation-based method for dynamical network reduction, together with its theoretical justification. To this end, we first extend the notion of the controllability Gramian to nonlinear systems from a statistical mechanics viewpoint, and show the validity through its application to controllability quantification. Then we show that, when the network is open to environmental noise, the newly proposed Gramian is equal to the covariance matrix of the uncontrolled dynamics. This equality brings about new insights into the relationship between controllability, simulation data, and stochasticity. This work is largely inspired by the path integral approach proposed by Kappen^28^. Although this concept is not directly used as a numerical procedure to solve the optimal control problem below, it is a key building block to prove the main result.

## Results

### Controllability function and Gramian.

Consider the nonlinear controlled dynamics:


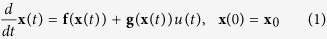


where *t* represents time, **x**(*t*) = [*x*_1_(*t*), …, *x*_*n*_(*t*)]^*T*^ and *u*(*t*) are the state and input variables, and smooth functions **f** and **g** describe the autonomous dynamics and the input effect, respectively. In (1), the initial state **x**_0_ is fixed, which affects both controllability determination and quantification. This can be arbitrarily chosen, although typically the initial state is fixed to a stable equilibrium in the conventional controllability quantification results of nonlinear systems^22^. Moreover, all the results in this paper hold for any probabilistic initial state (i.e., **x**_0_ is a random variable) and multi input cases as far as **x**_0_ is independent of the input noise below. For a final time *τ* > 0, the minimum control effort 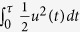 to achieve 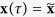 is referred to as a controllability function denoted by 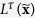. When the dynamics are linear, i.e., **f**(**x**) = **Ax** and **g**(**x**) = **B** with constant matrices **A**, **B** of compatible dimensions, the matrix 
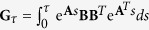
 is called the controllability Gramian. It is well known that 
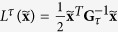
 provided **G**_*τ*_ is nonsingular when **x**_0_ = **0** 6,8,18. However, this definition of **G**_*τ*_ cannot straightforwardly be extended to nonlinear systems. Here, the controllability function and Gramian were introduced independently, and then a simple quadratic relation was shown. By changing our way of thinking, let us define **G**(*L*^*τ*^), which we call Gibbs Gramian, in terms of the Gibbs distribution associated with the given controllability function


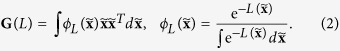


When 
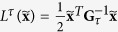
, the Gaussian integral formula shows **G**(*L*^*τ*^) = **G**_*τ*_. Therefore, this definition is consistent with the conventional one for linear dynamics. It should be noted that the definition of the controllability function does not assume linearity. Thus, this definition can readily be employed also for nonlinear cases. Another important feature is that we do not need to care about the reachability of each state. Even if some 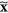 are not reachable by any finite energy input (e.g., linear dynamics for which **G**_*τ*_ is singular), 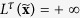 causes no problem in (2) because it simply leads to 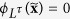. This means we can handle network dynamics that evolve on a specific domain due to the dynamics’ structure or physical constraints.

For linear systems with the initial state at the origin, eigenstructure of the controllability Gramian **G**_*τ*_ is useful for identifying directions in the state space that require small control energy to be reached. The Gibbs Gramian enjoys a similar property. Specifically, principle component analysis on **G**(*L*^*τ*^) reveals all effectively reachable directions. For instance, by setting 
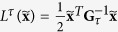
, we observe for the linear case that the principle eigenvector of **G**(*L*^*τ*^) = **G**_*τ*_ minimizes 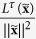, that is, the control effort divided by the squared distance takes its minimum value when the final state 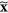 lies on the principle eigenvector. Another interpretation is that, of every possible direction, with a fixed control energy the state can be driven the furthest from the origin by driving it to a destination state that lies along the principle eigenvector. Interpretation for the nonlinear case has similarities with the linear case. Note that, for any unit vector **e**, large 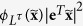 implies that a small energy input can be used (i.e., small 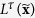, and consequently large 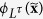) to place the state far from the origin along the direction of **e** (i.e., large 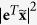) at the final time. Then, its spatial integral over all final states 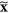 satisfies the following theorem, which readily follows from the equality 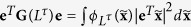:

*The integral* 
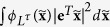
 *is maximized when* **e** *is the principal eigenvector of* **G**(*L*^*τ*^).

In this sense, the principal eigenvector of the Gibbs Gramian captures the direction along which the states can be reached furthest from the origin by a control effort that is small on average. In addition, it is trivial to change the reference point. For example, one can modify the definition as 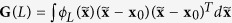 to evaluate the travelling distance instead of the distance from the origin. Similarly, the secondary, and further, eigenvectors enable us to characterize an effectively reachable subspace. See also the subsection entitled *Dimensionality reduction of nonlinear network dynamics* below for another interpretation in terms of the minimal projection error. The conclusion is that the Gibbs Gramian introduced **G**(*L*^*τ*^) in (2) is a proper extension of the conventional controllability Gramian **G**_*τ*_ for nonlinear dynamics.

### Stochasticity connects Gibbs Gramian and simulation data

**Figure 1:**
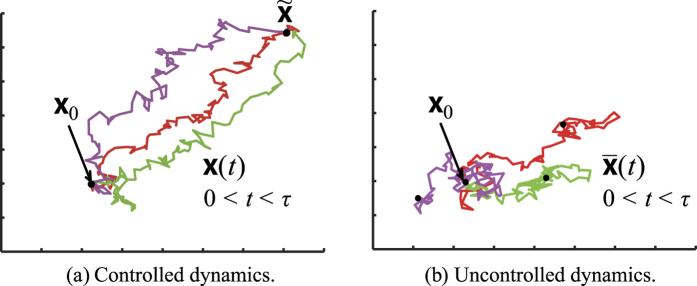
Typical behavior of controlled and uncontrolled dynamics open to environmental noise. (**a**) Sample paths of (3) controlled by a fixed feedback law that regulates 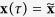. The corresponding control effort is measured by 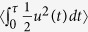, which is the average of 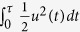 over these sample paths. Then, 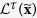 is the minimum of these average values over all such control laws. (**b**) Sample paths of the noise response 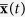 in (6).

For linear dynamics, **G**_*τ*_ is given as a solution to a linear matrix equation. On the other hand, the controllability function *L*^*τ*^ is given as a solution to a nonlinear partial differential equation for nonlinear dynamics^22^. Therefore, it is not realistic to compute *L*^*τ*^, and consequently **G**(*L*^*τ*^), even for small-scale cases. However, the situation drastically changes when the input is disturbed by random noise:


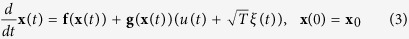


where *T* > 0 is a noise level or temperature, *ξ*(*t*) is white noise such that 〈*ξ*^2^(*t*)〉 = 1, and the expectation is taken over noise samples^29^,^30^. There are several theoretical results concerning the controllability determination of stochastic systems (e.g., approximate controllability^31^,^32^). In this paper, we define a *stochastic controllability function* 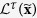 for the controllability quantification as


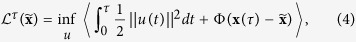


where the infimum is taken over all feedback control laws and we define Φ such that


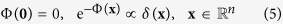


with the Dirac’s delta function *δ*. Then, the terminal cost is an alternative representation of the terminal boundary constraint 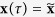, since 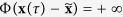 when 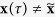. Therefore, 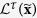 can be viewed as the minimum expected value of the control effort to regulate 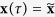; see Fig. 1a. Similarly to the deterministic case, we do not require the boundedness of 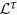. It should be emphasized that the resulting Gibbs distribution 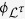 is not identical to 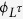, and depends on *T*. Next, we refer to the uncontrolled (*u*(*t*) = 0), but randomly excited dynamics 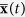 as noise response


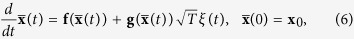


whose sample path is shown in Fig. 1b. The key finding in this paper is the following theorem, the proof of which is in the *Methods* section:

*The probability density function of* 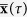 *is given by* 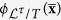
*, that is, the noise response* 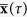 *obeys the Gibbs distribution associated with* 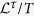.

This result means that the noise response data completely characterizes the minimum required input energy 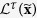 for each target state 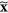. An intuitive reason for this nontrivial relation to hold is that the noise in (6) is added through the input channel. This type of noise is known to have an ability to search for the solution to a wide class of optimal control problems^28^. By this connection, the noise response data 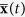 inherently contains information about the control energy minimization problem. Therefore, this bridges the gap to the controllability function that is defined via the minimum energy control input.

Note that the evaluation of 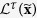 over the whole state space based on the density function estimation of 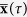 is still computationally intractable. However, in this paper, we focus on the Gramian induced by the stochastic controllability function, which is given by the spatial integral in (2), and is much easier to determine than the pointwise evaluation of 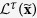. Actually, the theorem above yields the following equality for the *stochastic Gibbs Gramian* 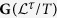:


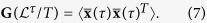


This equality tells us that the stochastic Gibbs Gramian can easily be calculated via Monte Carlo sampling of the uncontrolled dynamics open to environmental noise. Furthermore, both this computation and also the principle component analysis of 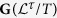 are efficiently implementable because various algorithms to achieve computational scalability exist for both Monte Carlo sampling (e.g., importance sampling) and matrix eigenvalue analysis. Thus, the novel equality (7) characterized in this paper leads to the first numerically tractable procedure to find an effectively reachable subspace of large scale nonlinear dynamics, when they are open to environmental noise, and is particularly useful for network dynamics for which only simulation algorithms, or time-series data collected in a noisy environment, are available.

### Dimensionality reduction of nonlinear network dynamics

The controllability quantification enables us to characterize subspaces that require a large control energy to be reached. By eliminating such subspaces, we can obtain a reduced order model, which is expected to well approximate the state trajectories as long as the input energy is not large. Actually, the dimensionality reduction of (mainly linear^18^,^33^) dynamical systems, which is helpful for understanding the hidden core mechanism, or to perform efficient numerical simulation, is an important application of the controllability quantification. In this section, we investigate two conceptually different nonlinear model reduction methods in the light of the Gibbs Gramian.

Let an integer *k*(<*n*) be the desired order of the reduced model and define the set of (*n* × *k*)-matrices **Π**_*k*_ = {***ρ*** : ***ρ***^*T*^***ρ*** = **I**} where **I** denotes the identity matrix. Suppose some ***ρ*** ∈ **Π**_*k*_ satisfies


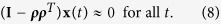


Then, the reduced state 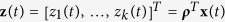 can approximately recover the original one by **x**(*t*) ≈ ***ρ*****z**(*t*). Hence, we expect the Galerkin projection given as 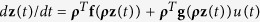 to be a good reduced order model of dynamics (1). In what follows, we focus on the problem of finding such a ***ρ***.

In computational physics, the Proper Orthogonal Decomposition (POD), or Karhunen-Loeve method, has a long history of intensive research^25^,^26^. This is a simulation-based model reduction method, and is widely used for the simulation of nonlinear large-scale dynamical systems as found in computational fluid dynamics and aerospace engineering. Suppose we replace the requirement (8) by 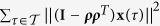 where the error is evaluated at multiple, given time instances 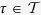. (This optimization criterion is equivalent to the maximal singular value of (**I** − ***ρρ***^*T*^) [**x**(*τ*_1_), **x**(*τ*_2_), …]). This is the fundamental idea of the POD, and is referred to as the method of snapshots. If we need to approximate only the autonomous system *d***x**(*t*)/*dt* = **f**(**x**(*t*)), this optimization is computationally tractable even for nonlinear large-scale dynamics, and the resulting Galerkin projection yields a satisfactory reduced model. However, when controlled dynamics (1) are of interest, we need to determine which input signal *u*(*t*) should be injected when collecting snapshots, because we cannot simulate the trajectories corresponding to all possible input signals. Many practically useful techniques, as well as theoretical analysis tools, for this have been developed; see^34^ and references therein. On the other hand, from a controllability quantification viewpoint, it is also reasonable to find ***ρ*** such that 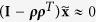 if 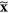 is reachable with a small energy input *u*(*t*). For this purpose, the Gramian-based model reduction for linear systems employs a ***ρ*** that maximizes Trace(***ρ*****G**_*τ*_***ρ***^*T*^). The Galerkin projection associated with this choice extracts effectively reachable subdynamics, in that the resulting projection eliminates a subspace on which 
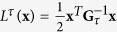
 is large. However, as mentioned at the beginning of the previous section, for nonlinear dynamics it is unrealistic to compute the controllability function *L*^*τ*^, which is no longer a quadratic form. This is the main reason why there have been no practical methods for the control-theoretic model reduction of general nonlinear large-scale systems^20^,^21^,^22^,^34^. This limited applicability shows a clear contrast to the POD. There are many results that attempt to solve optimal control problems by the POD^35^,^36^,^37^. However, the relation between the simulation-based and Gramian-based model reductions has not yet been fully understood.

The remainder of this section is devoted to forming a theory-bridge to connect these two model reduction approaches that were developed independently for similar purposes. Concerning the input selection for the POD, the impulse signals for the empirical Gramian^24^, or the sinusoidal signals for the frequency domain POD^21^,^25^, may be suitable for linear systems. Actually, the POD with these input signals is equivalent to the Gramian-based model reduction for linear systems [34, Chapter 5], [18, Section 9.1]. However, although it is technically easy to inject the same inputs for nonlinear systems, there is no solid justification for their use. An interesting solution is to choose white noise *ξ*(*t*) for the input signal, and minimize the snapshots’ ensemble average of the squared projection error, that is, 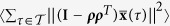. Note that (7) leads to


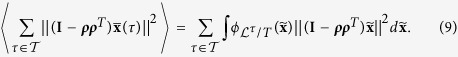


Therefore, the approximation error of the noise response data is equal to the projection error weighted by the Gibbs distribution associated with the stochastic controllability function 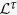. Consequently, the POD evaluates the projection errors on the trajectories that are realizable by a small control effort, without computing any minimum energy input. In this sense, the POD with noise response data can be regarded as an easily implementable nonlinear model reduction method that explicitly takes the controllability into account.

Note that (9) is equal to 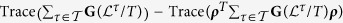. Therefore, the stochastic Gibbs Gramian-based reduction (the maximization of 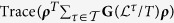) is equivalent to the best approximation of effectively reachable states (the minimization of the right-hand-side of (9)). This is another justification for the conclusion that the Gibbs Gramian is a proper extension of the conventional controllability Gramian.

Furthermore, equality (9) holds even for any nonlinear projection in the place of ***ρρ***^*T*^, although its optimization is nontrivial. In the case of linear systems, observability, a dual concept of the controllability, is also investigated, and often referred to as the balanced POD^34^. Extensions in this direction are currently under investigation.

## Discussion

The dynamics’ nonlinearity makes the controllability sensitive to *T*. This temperature dependency is discussed in this section. First, 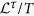 in (7) and (9) indicates that the input cost is inversely proportional to the noise level, that is, a less noisy (accurate) control channel is more expensive. In particular, as *T* → 0, the criterion 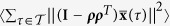 evaluates the error at the snapshots located almost on the trajectory of the autonomous system *d***x**(*t*)/*dt* = **f**(**x**(*t*)). Its interpretation from a controllability perspective is as follows: The input weight *T*^−1^ becomes unboundedly large, and consequently the states reachable with small control energy are limited to a small neighborhood around the autonomous trajectory (the noise is negligible because *T* → 0).

**Figure 2:**
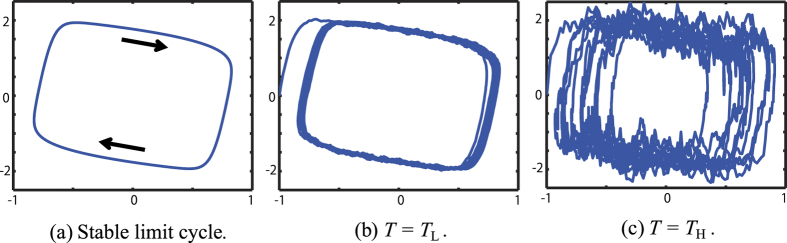
A phase portrait of the FitzHugh-Nagumo neuronal oscillator in the (*v*, *w*)-plane. (**a**) The stable limit cycle of the noise-free individual dynamics. (**b**) A sample path of **v**_1_(*t*) for *T* = *T*_L_. (**c**) A sample path of **v**_1_(*t*) for *T* = *T*_H_.

Next, we demonstrate the nontrivial effect of the noise level by means of a numerical example of *p* identical, coupled neuronal oscillators of the FitzHugh-Nagumo model. The individual neuron generates the stable limit cycle shown in Fig. 2a. The state variable of the *i*-th neuron is denoted by **v**_*i*_(*t*) = [*v*_*i*_(*t*) *w*_*i*_(*t*)]^*T*^, and the dimension of the entire system’s state **x**(*t*) = [**v**_1_(*t*)^*T*^ **v**_2_(*t*)^*T*^ … **v**_*p*_(*t*)^*T*^]^*T*^ is *n* = 2*p*. The dynamics of the *i*-th neuron, subject to the diffusive coupling with nonuniform intensity and external forcing, are given by


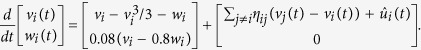


**Figure 3:**
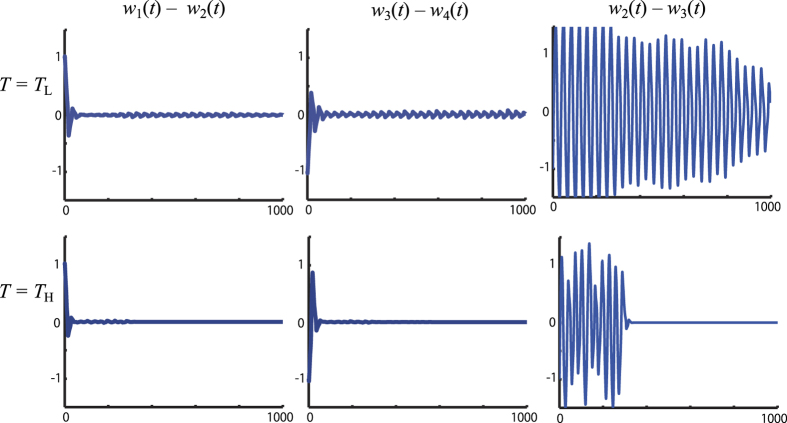
(De)Synchronization phenomena in a sample path. For both noise levels, (*w*_1_(*t*) − *w*_2_(*t*)) and (*w*_3_(*t*) − *w*_4_(*t*)) quickly decay due to the synchronization caused by the strong couplings. Synchronization is not observed in (*w*_2_(*t*) − *w*_3_(*t*)) for *T* = *T*_L_ because the coupling strength *η*_23_ is small. It shows a clear contrast to the quick noise induced synchronization for *T* = *T*_H_.

By using (7), we computed 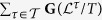 for 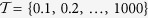 based on 1000 paths of the uncontrolled trajectories 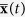 with *u*(*t*) = 0, and its normalized eigenvector **e**_*i*_ corresponding to the *i*-th largest eigenvalue *λ*_*i*_. Low (*T*_L_ = 0.05^2^) and high (*T*_H_ = 0.5^2^) noise levels are considered. Let *p* = 4 and 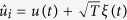 for all *i*, which means that only a common input is allowed. The symmetric coupling strengths *η_ij_*(=*η*_*ji*_) are given by *η*_12_ = *η*_34_ = 0.1, *η*_23_ = 0.005, and 0 for other pairs. The initial states are **v**_1_(0) = −**v**_3_(0) = [−1 0]^*T*^, **v**_2_(0) = −**v**_4_(0) = [0 2]^*T*^. For the uncontrolled trajectories 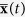 with *u*(*t*) = 0, apart from the fluctuation shown in Fig. 2, we observed the following 3 (de)synchronization phenomena with a high probability: (A) (**v**_1_ − **v**_2_) and (**v**_3_ − **v**_4_) quickly decayed due to their strong couplings, (B) (**v**_2_ − **v**_3_) decayed only slowly for *T* = *T*_L_ because their coupling is weak, (C) (**v**_2_ − **v**_3_) quickly decayed for *T* = *T*_H_ because noise-induced synchronization occurred^38^,^39^. See Fig. 3 for these phenomena observed in a sample path.

As explained in Table 1, **e**_1_ and **e**_2_ approximately span the subspace given by


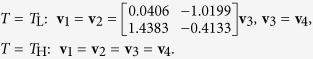


Recall that ***ρ*** = [**e**_1_ **e**_2_] minimizes (9) because 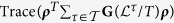 is maximized; see the previous section. Thus, the Galerkin projection onto this subspace extracts core subdynamics in the following two senses. First, from a POD perspective, this subspace best approximates the noise response data; see the left-hand-side of (9). This is confirmed by the fact that quick convergence to this subspace is nothing but the aforementioned (de)synchronization phenomena. Second, from a controllability perspective, this subspace best approximates the effectively reachable states; see the right-hand-side of (9). In other words, even if we apply the optimal feedback control, it is expensive to avoid the (de)synchronization phenomena.

This can also be understood from the structure of the dynamics. Concerning (A) and (C), since only the common input is allowed, the synchronization induced by the strong coupling and noise is difficult to prevent, independently of *T*. On the other hand, concerning the desynchronization in (B), even though some well designed entrainment signals exist^38^, they are not effective enough when the input weight *T*^−1^ is large. As observed in this example, controllability of highly nonlinear phenomena can be suitably captured from the noise-driven simulation data. Note that reduced order models for controlled complex networks obtained by the proposed method do not always allow such a simple interpretation. In other words, this method can extract nontrivial core dynamics purely from time series data.

In summary, we have proven that the noise response of the uncontrolled dynamics reveals the temperature-dependent controllability of general nonlinear network dynamics. This contribution consists of the following two achievements: One is a novel extension of the celebrated controllability Gramian for linear systems. To the author’s best knowledge, this is the first nonlinear extension of the controllability Gramian, which was introduced over half a century ago and played a central role in the development of modern control theory^5^. The second achievement is equality (7), which mathematically proves that, when the system is open to environmental noise, the newly introduced Gramian is equal to the covariance matrix of the noise response data. This result forms a theory-bridge connecting controllability quantification and time series data analysis. An important outcome is that the equality (9) yields an easily implementable method to a control-theoretic nonlinear model reduction problem for the first time. An extensive amount of noisy data is presently being gathered, and has been gathered to date, for a variety of uncontrolled systems. The equality (7) makes such data useful to glean insight into the modeling/controllability of controlled systems. We believe that this result can provide new methods and viewpoints in many research fields in view of the fact that much controllability related work is inspired by the pivotal contribution by Liu *et al.*^1^ For example, the condition number of the Gibbs Gramian should characterize the effect of nonlinearity on the network nonlocality analogously to the linear case^8^. Also, in view of the numerical simulation above, the relation between the noise effect and the connection topology of the dynamical complex networks^40^ can be analyzed. Furthermore, the fact that any uncontrolled nonlinear dynamics subject to environmental noise obey the Gibbs distribution associated with 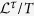, which is the minimum input energy divided by the temperature, suggests a nontrivial link to the *canonical distribution* that is used in statistical mechanics.

## Methods

For simplicity of exposition we let *T* = 1, but note that general results can be shown similarly. It is well known^28^ that the optimal value of the stochastic control problem in (4) satisfies 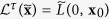 where the real scalar function 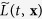 is the solution to the Hamilton-Jacobi-Bellman equation


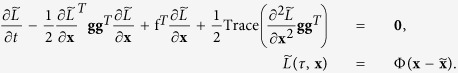


Next, the logarithmic transformation 
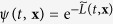
 yields the linear PDE


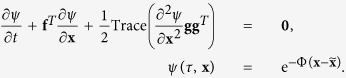


This form allows us to apply the Feynman-Kac formula^29^ to obtain


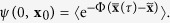


Based on 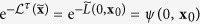 and (5), we have


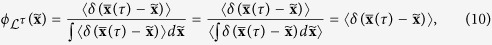


and consequently,


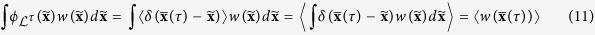


for an arbitrary smooth function *w*(**x**) defined on 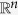, where we exchanged the order of expectation and spatial integral. The arbitrariness of *w*(**x**) in (11) means the probability density function of 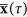 is given by 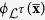. Finally, (11) with *w*(**x**) = **xx**^*T*^ yields (7).

## Additional Information

**How to cite this article**: Kashima, K. Noise Response Data Reveal Novel Controllability Gramian for Nonlinear Network Dynamics. *Sci. Rep.* **6**, 27300; doi: 10.1038/srep27300 (2016).

## Figures and Tables

**Table 1:** The eigenvectors of 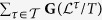 for the example.

	*T* = *T*_L_	*T* = *T*_H_
*ρ*_1_	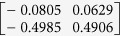	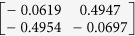
*ρ*_2_	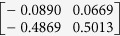	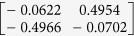
*ρ*_3_	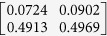	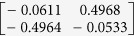
*ρ*_4_	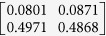	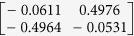

For both noise levels, *λ*_*i*_/*λ*_1_ < 0.15 for *i* = 3, 4, …, 8. The eigenvectors e_1_, e_2_ are given by 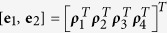 with *ρ*_*i*_ listed above. Based on the standard correlation analysis, we conclude that *ρ*_1_ ≈ *ρ*_2_, *ρ*_3_ ≈ *ρ*_4_, *ρ*_2_ _≉_ *ρ*_3_ for *T* = *T*_L_, and *ρ*_1_ ≈ *ρ*_2_ ≈ *ρ*_3_ ≈ *ρ*_4_ for *T* = *T*_H_.

## References

[b1] Liu Y.-Y., Slotine J.-J. & Barabási A.-L. Controllability of complex networks. Nature 473, 167–173 (2011). 21562557 10.1038/nature10011

[b2] Yuan Z., Zhao C., Di Z., Wang W.-X. & Lai Y.-C. Exact controllability of complex networks. Nat. Commun. 4, 2447 (2013). 24025746 10.1038/ncomms3447 PMC3945876

[b3] Power J. D. *et al.* Functional network organization of the human brain. Neuron 72, 665–678 (2011). 22099467 10.1016/j.neuron.2011.09.006 PMC3222858

[b4] Delpini D. *et al.* Evolution of controllability in interbank networks. Sci. Rep. 3, 1626 (2013). 23568033 10.1038/srep01626 PMC3620902

[b5] Kalman R. E. Mathematical description of linear dynamical systems. J. Soc. Ind. Appl. Math. Ser. A 1, 152–192 (1963).

[b6] Zhou K., Doyle J. C. & Glover K. K. Robust and optimal control (Prentice Hall, 1996).

[b7] Yan G., Ren J., Lai Y. C., Lai C. H. & Li B. Controlling complex networks: How much energy is needed? Phys. Rev. Lett. 108, 218703 (2012). 23003312 10.1103/PhysRevLett.108.218703

[b8] Sun J. & Motter A. E. Controllability transition and nonlocality in network control. Phys. Rev. Lett. 110, 208701 (2013). 25167459 10.1103/PhysRevLett.110.208701

[b9] Menichetti G., Dall’Asta L. & Bianconi G. Network controllability is determined by the density of low in-degree and out-degree nodes. Phys. Rev. Lett. 113, 078701 (2014). 25170736 10.1103/PhysRevLett.113.078701

[b10] Nepusz T. & Vicsek T. Controlling edge dynamics in complex networks. Nat. Phys. 8, 568–573 (2012).

[b11] Posfai M., Liu Y.-Y., Slotine J.-J. & Barabási A.-L. Effect of correlations on network controllability. Sci. Rep. 3, 1067 (2013). 23323210 10.1038/srep01067 PMC3545232

[b12] Yuan Z., Zhao C., Di Z., Wang W.-X. & Lai Y.-C. Exact controllability of multiplex networks. New J. Phys. 16, 103036 (2014).

[b13] Zhao C., Wang W.-X., Liu Y.-Y. & Slotine J.-J. Intrinsic dynamics induce global symmetry in network controllability. Sci. Rep. 5, 8422 (2015). 25672476 10.1038/srep08422 PMC4325315

[b14] Cowan N. J., Chastain E. J., Vilhena D. A., Freudenberg J. S. & Bergstrom C. T. Nodal dynamics, not degree distributions, determine the structural controllability of complex networks. PLoS One 7, e38398 (2012). 22761682 10.1371/journal.pone.0038398 PMC3382243

[b15] Wang W. X., Ni X., Lai Y. C. & Grebogi C. Optimizing controllability of complex networks by minimum structural perturbations. Phys. Rev. E 85, 026115 (2012). 10.1103/PhysRevE.85.026115 22463287

[b16] Whalen A. J., Brennan S. N., Sauer T. D. & Schiff S. J. Observability and controllability of nonlinear networks: The role of symmetry. Phys. Rev. X 5, 011005 (2015). 10.1103/PhysRevX.5.011005 PMC6234006 30443436

[b17] Cornelius S. P., Kath W. L. & Motter A. E. Realistic control of network dynamics. Nat. Commun. 4, 1942 (2013). 23803966 10.1038/ncomms2939 PMC3955710

[b18] Antoulas A. C. Approximation of Large-Scale Dynamical Systems (SIAM, 2005).

[b19] Schilders W. H. A., Vorst H. A. V. D. & Rommes J. (eds) Model Order Reduction: Theory, Research Aspects and Applications (Springer-Verlag, 2008).

[b20] Besselink B., van de Wouw N., Scherpen J. M. A. & Nijmeijer H. Model reduction for nonlinear systems by incremental balanced truncation. IEEE Trans. Autom. Contr. 59, 2739–2753 (2014).

[b21] Astolfi A. Model reduction by moment matching for linear and nonlinear systems. IEEE Trans. Autom. Contr. 55, 2321–2336 (2010).

[b22] Scherpen J. Balancing for nonlinear systems. Syst. Contr. Lett. 21, 143–153 (1993).

[b23] Zuazua E. Averaged control. Automatica 50, 3077–3087 (2014).

[b24] Lall S., Marsden J. E. & Glavaški S. A subspace approach to balanced truncation for model reduction of nonlinear control systems. Int. J. Robust Nonlin. Contr. 12, 519–535 (2002).

[b25] Willcox K. & Peraire J. Balanced model reduction via the proper orthogonal decomposition. AIAA J. 40, 2323–2330 (2002).

[b26] Kunisch K. & Volkwein S. Galerkin proper orthogonal decomposition methods for a general equation in fluid dynamics. SIAM J. Numer. Anal. 40, 492–515 (2002).

[b27] Hahn J., Edgar T. F. & Marquardt W. Controllability and observability covariance matrices for the analysis and order reduction of stable nonlinear systems. J. Process Contr. 13, 115–127 (2003).

[b28] Kappen H. J. Linear theory for control of nonlinear stochastic systems. Phys. Rev. Lett. 95, 200201 (2005). 16384034 10.1103/PhysRevLett.95.200201

[b29] Karatzas I. & Shreve S. E. Brownian Motion and Stochastic Calculus No. 113 in Graduate texts in mathematics, 2nd edn (Springer, 1998).

[b30] Masuda N., Kawamura Y. & Kori H. Collective fluctuations in networks of noisy components. New J. Phys. 12, 093007 (2010).

[b31] Buckdahn R., Quincampoix M. & Tessitore G. A characterization of approximately-controllable linear stochastic differential equations. In Stochastic Partial Differential Equations and Applications–VII 53–60 (Taylor & Francis, 2006).

[b32] Mahmudov N. I. & Zorlu S. Controllability of non-linear stochastic systems. Int. J. Contr. 76, 95–104 (2003).

[b33] Ishizaki T., Kashima K., Imura J.-I. & Aihara K. Model reduction and clusterization of large-scale bidirectional networks. IEEE Trans. Autom. Contr. 59, 48–63 (2014).

[b34] Holmes P., Lumley J. L., Berkooz G. & Rowley C. W. Turbulence, coherent structures, dynamical systems and symmetry. Cambridge monographs on mechanics, 2nd ed (Cambridge University Press, 2012).

[b35] Kunisch K., Volkwein S. & Xie L. HJB-POD-based feedback design for the optimal control of evolution problems. SIAM J. Appl. Dyn. Syst. 3, 701–722 (2004).

[b36] Kunisch K. & Xie L. POD-based feedback control of the burgers equation by solving the evolutionary HJB equation. Comput. Math. Appl. 49, 1113–1126 (2005).

[b37] Hinze M. & Volkwein S. Proper orthogonal decomposition surrogate models for nonlinear dynamical systems: Error estimates and suboptimal control. In Dimension Reduction of Large-Scale Systems vol. 45 of Lecture Notes in Computational Science and Engineering 261–306 (Springer Berlin Heidelberg, 2005).

[b38] Harada T., Tanaka H. A., Hankins M. J. & Kiss I. Z. Optimal waveform for the entrainment of a weakly forced oscillator. Phys. Rev. Lett. 105, 088301 (2010). 20868133 10.1103/PhysRevLett.105.088301

[b39] Teramae J. N. & Tanaka D. Robustness of the noise-induced phase synchronization in a general class of limit cycle oscillators. Phys. Rev. Lett. 93, 204103 (2004). 15600929 10.1103/PhysRevLett.93.204103

[b40] Ren J., Wang W. X., Li B. & Lai Y. C. Noise bridges dynamical correlation and topology in coupled oscillator networks. Phys. Rev. Lett. 104, 058701 (2010). 20366800 10.1103/PhysRevLett.104.058701

